# Rehabilitation to Improve Outcomes after Cervical Spine Surgery: Narrative Review

**DOI:** 10.3390/jcm13185363

**Published:** 2024-09-10

**Authors:** Tomoyoshi Sakaguchi, Ahmed Heyder, Masato Tanaka, Koji Uotani, Toshinori Omori, Yuya Kodama, Kazuhiko Takamatsu, Yosuke Yasuda, Atsushi Sugyo, Masanori Takeda, Masami Nakagawa

**Affiliations:** 1 Department of Rehabilitation, Okayama Rosai Hospital, 1-10-25 Chikkomidorimachi, Minami Ward Okayama, Okayama 702-8055, Japan; tomoyoshi0127@gmail.com (T.S.); kazuhikopt0803@gmail.com (K.T.); kyushudanji19861007@gmail.com (Y.Y.); ot2632nakagawa@gmail.com (M.N.); 2 Department of Orthopedic Surgery, Okayama Rosai Hospital, 1-10-25 Chikkomidorimachi, Minami Ward Okayama, Okayama 702-8055, Japan; dr.a.heydar@gmail.com (A.H.); coji.uo@gmail.com (K.U.); sooseizi0402@yahoo.co.jp (T.O.); ykodama314@gmail.com (Y.K.); 3 Department of Rehabilitation, Spinal Injuries Center, 550-4 Igisu, Fukuoka 820-8508, Japan; a.sugyo@gmail.com; 4 Department of Rehabilitation, Kansai Rosai Hospital, 3-1-69 Inabasou, Amagasaki City 660-8511, Japan; pttake@yahoo.co.jp

**Keywords:** cervical spine, rehabilitation, physiotherapy, muscle exercise

## Abstract

Purpose: The increasing elderly patient population is contributing to the rising worldwide load of cervical spinal disorders, which is expected to result in a global increase in the number of surgical procedures in the foreseeable future. Cervical rehabilitation plays a crucial role in optimal recovery after cervical spine surgeries. Nevertheless, there is no agreement in the existing research regarding the most suitable postsurgical rehabilitation program. Consequently, this review assesses the ideal rehabilitation approach for adult patients following cervical spine operations. Materials and Methods: This review covers activities of daily living and encompasses diverse treatment methods, including physiotherapy, specialized tools, and guidance for everyday activities. The review is organized under three headings: (1) historical perspectives, (2) patient-reported functional outcomes, and (3) general and disease-specific rehabilitation. Results: Rehabilitation programs are determined on the basis of patient-reported outcomes, performance tests, and disease prognosis. CSM requires strengthening of the neck and shoulder muscles that have been surgically invaded. In contrast, the CCI requires mobility according to the severity of the spinal cord injury and functional prognosis. The goal of rehabilitation for CCTs, as for CCIs, is to achieve ambulation, but the prognosis and impact of cancer treatment must be considered. Conclusions: Rehabilitation of the cervical spine after surgery is essential for improving physical function and the ability to perform daily activities and enhancing overall quality of life. The rehabilitation process should encompass general as well as disease-specific exercises. While current rehabilitation protocols heavily focus on strengthening muscles, they often neglect the crucial aspect of spinal balance. Therefore, giving equal attention to muscle reinforcement and the enhancement of spinal balance following surgery on the cervical spine is vital.

## 1. Introduction

The most critical cervical diseases that require rehabilitation are cervical spondylotic myelopathy (CSM), cervical spinal cord injury (CCI), and cervical spinal cord tumor (CCT). CSM is an age-related progressive degenerative disease of the spine that results in cervical spinal cord dysfunction [1,2]. CSM is the leading cause of cervical myelopathy in individuals aged 55 or older [3]. The onset of signs and symptoms is often gradual, and they may include, in addition to urologic symptoms, a loss of hand dexterity, muscle weakness, joint stiffness, spasticity in the extremities, and gait abnormalities [4,5,6].

The incidence of cervical spine cord injury (CCI) is estimated at 13 per 100,000 people, with traffic crashes, falls, self-inflicted injuries, and occupational accidents being the most common causes worldwide [7]. CCI is a devastating neurological state that results in physical dependency, morbidity, psychological stress, and financial burden [8]. Rehabilitation for CCI patients is essential to prevent complications such as decubitus ulcers, joint contraception, and acute muscle atrophy and to improve independent mobilization in chronic patients [9].

The increased possibility of early detection of cervical cord tumors (CCTs) and advances in tumor management have improved the life expectancy of these patients [10]. As many as 85% of CCT patients may present with metastatic spinal cord compression. Rehabilitation should consider the body’s ability due to oncologic treatment, but it is essential to shorten the hospital stay and return to daily activities [10,11]. In the multidisciplinary team approach, when the rehabilitation of CCTs is combined with improved medical, radiological, and surgical treatment, patient and family efforts are consolidated, function is enhanced, and complications from neurologic damage are prevented [12]. Recently, an increase in the number of reports regarding rehabilitation following cervical spine surgery has been reported [13,14]. However, there has been no comprehensive review of postoperative rehabilitation of CSM, CCI, and CCT patients. We believe that this would be an essential report for healthcare providers who perform rehabilitation after cervical spine surgery.

This paper aims to thoroughly examine rehabilitation’s historical background, frequently employed patient-reported outcome evaluation techniques, modern viewpoints on spinal rehabilitation following surgery, and methods for integrating rehabilitation into the recovery journey after cervical spine procedures.

## 2. Materials and Methods

A thorough literature investigation focused on rehabilitation and patient-reported outcome assessment methods following cervical spine operations. Our sources included PubMed-indexed, peer-reviewed journals, clinical data, and case studies that involved terminologies such as cervical spine surgery, rehabilitation, and assessment. The search terms we used to access the database included “Cervical spine”, “Rehabilitation”, “Surgery”, “Evaluation”, “CSM”, “CCI”, and “CCT”. We focused on papers published after 1990 when cervical rehabilitation became more widespread [15]. We eliminated case reports, technical notes, review articles, and publications with an impact factor of less than one from our analysis.

## 3. Results

### 3.1. Historical Review of Rehabilitation for Cervical Diseases

From 1914 to 1924, this period was the pre-revenue phase of rehabilitation. Rehabilitation was aimed mainly at occupational rehabilitation because many soldiers were injured during World War I [16]. Physical and occupational therapies have emerged as crucial adjuncts of surgical practice, particularly for patients with orthopedic injuries. These therapies have been pioneered by experts such as R. Tait McKenzie and George Deaver. Over a decade later, the medicine of spinal cord injury became a well-known field due to the high number of casualties during World War II [17]. Treating spinal cord injuries requires a multidisciplinary team led by a physiatrist, occupational therapist, physiotherapist, psychologist, social worker, speech therapist, and other specialist consultants as needed. In 1944, the British Council for Rehabilitation defined “rehabilitation” as “the whole range of services from the time of the onset of the individual’s disability to the point at which he is restored to normal activity or the nearest possible approach to it” [18]. Furthermore, the World Health Organization (WHO) termed rehabilitation activities of daily living (ADL) improvement in 1968 [19]. Since then, the importance of rehabilitation has increased widely worldwide.

George Engel’s groundbreaking biopsychosocial model arose from a lack of satisfaction with the biomedical model of illness. His novel model highlighted the dualistic and dynamic perspective of human experience, signifying the mutual influence of the mind and body [20]. This allows the rehabilitation framework to encompass biomedical and social, psychological, and behavioral aspects of illness.

In approximately 1980, rehabilitation focused on patients’ quality of life (QOL) because of the increasing number of elderly patients and the reduced risk of falls [21]. Despite the increasing incidence of traffic accidents and residual patient neck symptoms, there is no clear rehabilitation protocol. The Ontario Protocol for Traffic Injury Management (OPTIMa) Collaboration suggested in 2016 that clinicians should take into account supervised strengthening exercises and structured patient education to manage patients with acute (less than three months) cervical radiculopathy [22]. However, new approaches to rehabilitation, such as combined physical and psychological treatments, have been proposed, as new concepts of psychological factors have been considered [23,24] (Table 1).

### 3.2. Different Types of Rehabilitation

The goal of rehabilitation following spinal surgical interventions is to increase physical, psychological, and social functioning, accelerate recovery, prevent and treat complications, address residual symptoms, and treat associated diseases [25]. Patients may experience reduced neck motion due to multiple factors, such as fusion, pain, and immobilization, in the immediate postoperative period, resulting in severe muscle atrophy and symptom persistence after surgery [26,27]. Postsurgical rehabilitation may be recommended by spine surgeons, general practitioners, physical therapists, chiropractors, and occupational therapists, depending on the individual’s needs. Table 2 shows examples of postsurgical rehabilitation interventions.

Examples of postsurgical rehabilitation interventions are shown in Table 2.

## 4. Patients-Reported Outcome (PRO) Measures

The PRO measures are progressively being used to assess value-based care. PROs are any reports from patients regarding their health [38]. They are particularly useful for subjective outcomes, such as functional health status, health perceptions, and quality of life (QOL) [38]. PROs are widely used in clinical settings [39,40]. A critical concept in PRO analysis is the minimal clinically significant difference (MCID). The slightest change in treatment outcome that patients consider significant is known as the MCID. This represents the threshold at which a patient perceives a meaningful improvement in their condition due to the treatment [41]. Another essential concept is the patient-acceptable symptom state (PASS), which is the score of a PRO scale that indicates that patients perceive themselves as being in a satisfactory or healthy condition [42]. A systematic review by Issa et al. reported the postoperative MCID for cervical spine disorders [42]. The reported MCID values for cervical spine surgery are shown in Table 3.

### 4.1. Neck Disability Index (NDI)

The NDI was developed in 1991 [49]. It is the most widely utilized patient-reported outcome (PRO) measure internationally for postoperative patients with cervical spine injuries [50]. The NDI evaluates the degree of disability caused by neck pain. A modified version of the NDI was introduced by Takeshita et al. in 2013, which included the phrase “because of neck pain” in the phrase “because of neck pain or numbness in the arm” [51]. The Cronbach’s alpha values for the original and modified NDIs were excellent, at 0.92 and 0.89, respectively [51].

### 4.2. Japanese Orthopedic Association Cervical Myelopathy Evaluation Questionnaire (JOACMEQ)

The JOACMEQ serves as a patient-reported outcome instrument for assessing cervical myelopathy, which was developed in 2007 [52]. The assessment consists of 24 items and encompasses five key areas: functionality of the cervical spine, upper limbs, and lower limbs, bladder control, and overall life quality (QOL). The scores span from 0 to 100 points, with more significant numbers signifying more favorable circumstances. Recent studies have translated and utilized the JOACMEQ in various countries and demonstrated strong internal consistency, with Cronbach’s alpha values ranging from 0.88 to 0.91 [53,54].

### 4.3. Spinal Cord Injury–Quality of Life (SCI-QOL)

The SCI-QOL measurement was initially designed to address the deficiency of ordinary PRO available for the clinical management of patients with SCI. It assesses spinal cord-injured patients’ medical, functional, and psychological outcomes [55,56]. The SCI-QOL is comprised of 22 items and evaluates four key areas: (1) physical and medical well-being, (2) psychological health, (3) community engagement, and (4) physical capabilities [56]. The SCI-QOL has been related to the subjective effects of pressure ulcers, anxiety, and depression, with excellent reliability reported [57,58,59]. A shortened version of the SCI-QOL was also reported, with a Cronbach’s α value of 0.89, demonstrating good internal consistency reliability [60].

### 4.4. Disabilities of Arm Shoulder and Hand (DASH)

The DASH scoring system was developed to evaluate upper limb outcomes since it considers all parts of the upper limb as a single functional unit [61]. For patients with cervical spine disorders, pain and the restoration of upper extremity function are the highest priorities [62]. DASH is a significant postoperative upper extremity PRO for cervical spine surgery because it assesses upper extremity function not captured by other cervical spine-specific assessments [63,64]. Quick DASH, with fewer questions than the original DASH, was developed and was reliable, with a Cronbach’s alpha value of 0.92 [65]. Several studies using Quick DASH in patients with CSM have reported that it is related to NDI scores and upper extremity pain [66,67].

### 4.5. Swallowing-Related Quality of Life (SWAL-QOL)

The SWAL-QOL is used to assess dysphagia after anterior cervical spine surgery (ACSS) [68,69]. The SWAL-QOL comprises 44 items and encompasses ten domains (social functioning, sleep, fatigue, mental health, communication, burden, fear, food selection, eating duration, and eating desire); a better patient quality of life could be expected with higher scores [70,71]. It has been reported that 10.8% to 50.2% of patients after ACSS will experience dysphagia during the first postoperative year, with 4.8% having severe dysphagia [72,73]. Recently, an abridged version of the SWAL-QOL, which reduces the number of questions to 14 and is designed explicitly for post-ACSS, has been reported [74].

## 5. Physical Performance Tests

Hand clumsiness and gait disturbances are characteristic clinical manifestations of cervical spinal cord disease [75,76,77]. Patients with cervical myelopathy have locomotive syndrome, a condition in which motor function is impaired [78]. Patients with impaired motor function are still at high risk of falling even after surgery [79]. Although muscle strength is preserved, these patients have sensory ataxia and reduced proprioception [80]. Assessing physical function before and after surgery is essential to establish a rehabilitation program. The commonly used physical examinations include the following.

### 5.1. Ten-Second Grip and Release Test (10s-G&R) (Figure 1)

Ten-second G&R counts the number of times a finger is performed in 10 s by grasping and opening each finger on one side as quickly as possible [81]. Age-specific cutoff values have been reported for the G&R because the G&R is affected by age. CSM is generally suspected when the number of grips and releases is below the cutoff value [82]. A 10 s G&R of 17–20 cycles in CSM patients is classified as mild hand dexterity impairment, 14–16 cycles as moderate impairment, and 13 cycles or less as severe impairment, indicating increased disease severity and decreased quality of life [83].

**Figure 1:**
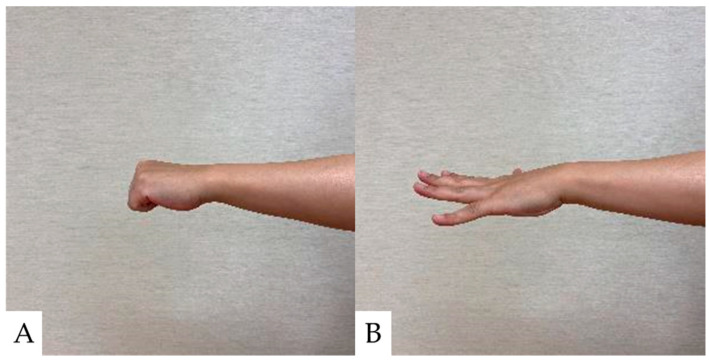
Ten-second Grip and Release Test. (**A**): Grip; (**B**): Release. A 10 s G&R of less than 20 times G&R is classified as hand dexterity impairment.

### 5.2. Capabilities of Upper Extremity Test (CUE-T) 

A patient-specific upper extremity function assessment method for CCI patients has been reported to have good validity, reliability, and responsiveness [84,85]. CUE-T is characterized by its ability to quantify functional limitations specific to spinal cord injury tetraplegia without needing a special evaluation kit. This consists of coarse movements to check one-handed movements, including reaching in each direction, two-handed movements for push-ups and lifting weights, and skillful movements to check grasping, pinching, and manipulation of objects performing wrist joint, index finger, and thumb movements [86].

### 5.3. Foot Tapping Test (FTT) (Figure 2)

The FTT is a method used to assess the speed at which the patients can flex and extend their toes constantly for ten seconds while their heels are kept on the ground (Figure 1). This test measures the speed of voluntary limb movements in patients with degenerative compression myelopathy [87]. The FTT also correlates with the 30 m walk test and is effortless to execute, as it can be accomplished while the patient sits in a chair [88]. Patients with myelopathy exhibited a significantly reduced mean FTT of 23.8 ± 7.2, compared to the 31.7 ± 6.4 observed in healthy individuals, with a decline noted as age increased [87]. Additionally, the FTT score significantly correlated with lower extremity motor function, as measured by the modified JOA score and the grip and release test [87].

**Figure 2:**
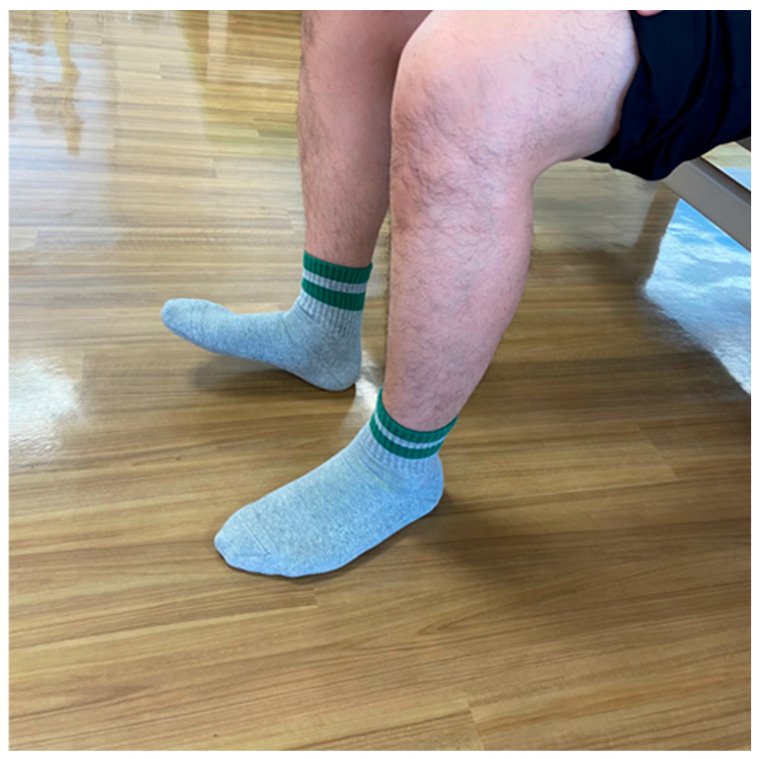
Foot Tapping Test.

### 5.4. The Brief BESTest (Table 4)

The Brief BESTest is a modified version of the BESTest [89], with six sections assessing biomechanical constraints, stability limits, anticipatory postural adjustments, postural responses, sensory orientation, and gait stability [90]. In a study on the psychometric properties of various balance assessment measures, the Brief BESTest was the most effective in assessing balance ability in CSM patients [89]. Patients with CSM are at a greater risk of falling if their Brief BESTest score is below eight, and a walking aid is recommended if the score is nine or lower [91].

**Table 4:** The Brief BESTest.

I. Biomechanical Constraints	II. Stability Limits	III. Anticipatory Postural Adjustments	IV. Postural Responses	V. Sensory Orientation	VI. Stability in Gait
1. Hip/trunk lateral strength	2. Functional reach forward	3. Stand on one leg (left and right)	4. Compensatory stepping correction, lateral (left and right)	5. Stance on foam, Eyes closed	6. Timed “Get Up & Go” Test

### 5.5. Walking Index for Spinal Cord Injury (WISCI II)

WISCI II is the most commonly utilized scale of walking ability [92,93,94]. This scale consists of 21 levels arranged in a hierarchy, assessing an individual’s capacity to traverse a 10 m distance on a level, unobstructed surface. The scoring system spans from 0, indicating an inability to stand or engage in assisted walking, to 20, representing the ability to walk 10 m independently without any assistive devices, orthoses, or physical support [95]. However, the WISCI score does not consider walking speed or gait quality. The assessment of participants is based on their performance in a controlled testing setting rather than considering their preferred walking habits in everyday situations or at their residences [95]. Nevertheless, the WISCI II score significantly correlates with the functional independence measure, walking speed, 6 min walking distance, lower extremity motor score, and Berg balance scale [96].

### 5.6. Trunk Control Test (TCT)

Individuals with spinal cord injuries (SCIs) cannot often sit unsupported due to paralysis and sensory loss, making trunk control an essential form of gross motor activity for those with paraplegia who perform most daily activities from a seated position. According to Anderson and colleagues [97], more than 60% of people with SCI and tetraplegia considered core stability combined with arm and hand function to be their highest priority for enhancing their overall quality of life. The TCT is a standard assessment tool for evaluating trunk function in SCI patients [98]. The assessment encompasses evaluations of seated stability and examinations of more intricate tasks associated with trunk control, including trunk flexion, extension, and rolling movements. Studies have demonstrated that the TCT possesses prognostic validity, as it can forecast walking ability and autonomy in SCI patients 12 months following their injury [99,100].

## 6. Active of Daily Living (ADL) Outcome Measures

Rehabilitation medicine involves assessing alterations in daily living (ADL) activities due to physical, mental, and functional decline and structural abnormalities caused by disease. Medical care attempts to restore function and adjust the environment to improve ADLs. Assessing ADLs is essential for understanding the patient’s status and determining the effectiveness of treatment. The comprehensive and disease-specific ADL assessment indicators are listed below. 

### 6.1. Spinal Cord Independence Measure (SCIM)

The SCIM comprises four areas of function: self-care, breathing, sphincter management, and mobility. It is rated on a 17-item scale out of 100, with higher scores indicating more independent ADLs. It is an SCI-specific ADL assessment index that evaluates important ADL items for patients with SCI, such as bed and decompression movements. Version IV is the latest version of the report [101]. Cronbach’s alpha value of 0.96 for the SCIM IV has been reported with excellent reliability and validity [102].

### 6.2. Barthel Index (BI)

The BI is a commonly used and validated measure to assess a patient’s activities of daily living and functional ability [103,104]. It consists of 10 items, including mobility, self-care, and toileting, with total scores ranging from 0 (full assistance) to 100 (independent). BI assesses “ADLs that can be performed” [105]. In a retrospective comparative study, Zhang et al. [106] compared the BI for different SCI levels at hospital admission and discharge. They reported that the BI improved from 24.7 to 52.7 points for patients with cervical SCI, 19.3 to 56.3 points for patients with thoracic SCI, and 18.1 to 67 points for patients with lumbar SCI. Additionally, the BI did not correlate with the surgical time, rehabilitation interval, or mean hospital stay for patients with spinal segment injuries.

### 6.3. Functional Independence Measure (FIM)

The FIM is the most commonly utilized ADL assessment. The FIM consists of two major items: motor and cognitive. The motor items are linked to self-care, toileting, transferring, and mobility activities, whereas the cognitive items are linked to communication and social awareness. It assesses a patient’s independence on a scale of 18–126 points on 18 items [107]. Unlike BI, it evaluates the ADLs that are being performed. It is often used for ADLs in patients with SCI, but it has recently been used to assess ADLs in patients with SCT and CSM [108,109,110]. The FIM is easy to use as a general ADL assessment tool but difficult for patients with SCI. The FIM does not adequately capture functional changes because it fails to assess turning over, getting up, and preventing pressure injuries, which are essential for patients with SCI.

## 7. Cervical Cord Injury Height Level and Severity Assessment Index

Severe injuries to the spinal cord often result in motor paralysis and sensory deficits in the extremities and trunk below the level of the injury. Moreover, the autonomic nervous system may lose its function. Accurate assessment of paralysis and deficits is crucial for setting rehabilitation goals and developing a suitable program. Notably, in tetraplegic patients, various functional abilities could be acquired through rehabilitation, even within the same spinal cord segment. It is also imperative to determine the prognosis for mobility in patients with spinal cord injuries.

### 7.1. International Standards for Neurological Classification of Spinal Cord Injury (ISNCSCI) and American Spinal Injury Association Impairment Scale (AIS) for Neurological Evaluation of SCI Patients

The ISNCSCI and AIS are the most broadly accepted systems for examining and classifying sensory and motor impairments in patients with spinal cord injuries. The ISNCSCI enables consistent and precise communication between researchers, clinicians, and patients. The information obtained from this system is utilized to develop personalized rehabilitation programs, predict patient prognosis, document recovery, and assess the effectiveness of interventions. The first edition of the ISNCSCI, the Standards for the Neurologic Classification of Spinal Cord Injury, was published by the ASIA in 1982 [111] and updated in 2019 [112]. The National Institute for Neurological Disorders and Stroke (NLI) score and AIS score can be determined from the sensory and motor scores. In a prospective analysis of 600 patients conducted by Scivoletto et al. [113], the MCIDs for the motor and sensory scores were 4.48 and 5.19, respectively. The MCIDs for the upper extremity motor score (UEMS) and lower extremity motor score (LEMS) were 2.72 and 3.66, respectively [113]. It is crucial to perform an accurate evaluation according to the International Standards for Neurological Classification of SCIs developed by the ASIA when planning a physical therapy program. This evaluation is clinically beneficial and essential for international research activities because it allows for the quantitative assessment and analysis of physical function.

### 7.2. International SCI Datasets

A comprehensive collection of common data elements (CDEs) for Spinal Cord Injury Clinical Research and Clinical Trials has been released by the National Institute of Neurological Disorders and Stroke (NINDS). The release set also included report forms and expert recommendations. This comprehensive set of CDEs is considered a valuable resource for clinical researchers who desire to use standardized data collection methods when performing new clinical studies. The set covers more than fifteen neurological disaster areas. Many ISCoS International SCI datasets have been incorporated in whole or in part into the NINDS SCI CDEs and recommendations. The NINDS CDEs and the ISCoS International SCI datasets are readily available through the NINDS CDE Project website [114]. Translations of the International SCI datasets are encouraged, but the recommendations in the publication must not be modified [115]. The NINDS SCI CDEs and the expert recommendations have incorporated numerous ISCoS International SCI datasets, either in full or partially. These resources are easily accessible through the NINDS CDE Project website [114]. While translations of the International SCI datasets are encouraged, the recommendations in the publication must remain unmodified.

## 8. Postoperative Physical Therapy for Cervical Surgery 

The need for surgical intervention in patients with cervical pain and neuropathies that do not respond to conservative treatment is increasing [25,116]. Anterior cervical discectomy and fusion are currently among the most common surgical procedures for the cervical spine [25,26,117]. The efficacy of cervical spine surgery for treating cervical nerve root pain is good, but its effect on neck function is unknown [25,118]. During the immediate postoperative period, cervical fusion decreases range of motion, pain, and muscle weakness [26,27,117]. Atrophy and deconditioning of cervical muscle function do not resolve spontaneously and persist over time [27,117,119]. Therefore, a structured postoperative rehabilitation program, including endurance exercises, isometric strengthening, stretching, neck and shoulder function, and aerobic exercise, is recommended to improve postoperative cervical spine dysfunction and neuropathy [27,116]. Compared with standard treatment, a structured therapeutic exercise program combined with a cognitive behavioral protocol improves neck disability, pain intensity, patient satisfaction, and patient anxiety after surgery [27,116,118]. It is necessary to establish a rehabilitation program that considers the functional and life prognoses of patients with spinal cord injury (SCI) and spinal cord tumors (SCTs). The following is a general postoperative rehabilitation plan for the cervical spine.

### 8.1. Neck and Shoulder Muscle Strengthening

**Figure 3:**
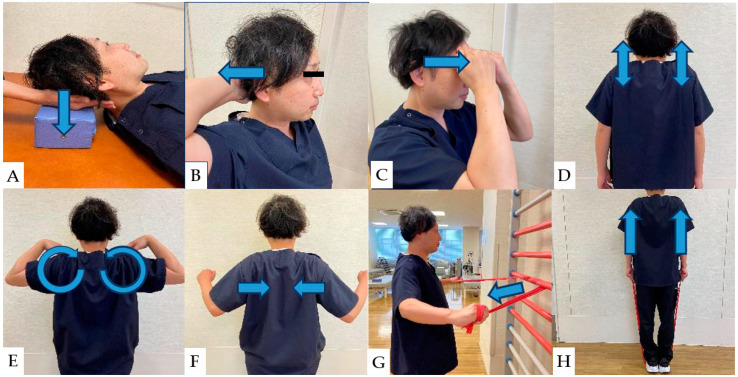
(**A**): Isometric contraction of the extensor muscles of the neck. (**B**): Self-isometric exercise of neck extensor muscles. (**C**): Self-isometric exercise of neck flexor muscles. (**D**): Scapular elevation exercises. (**E**): Scapular rotation exercises. (**F**): Scapular adduction exercises. (**G**): Resistance exercise of the middle trapezius muscle. (**H**): Resistance exercise of the upper trapezius muscle.

Strengthening of deep neck muscles is associated with improvements in NDI scores and neck and upper extremity pain [118]. Strengthening the deep neck muscles begins with nonresistance exercises and moves to isometric and resistance exercises [118]. Previous studies demonstrated a strong relationship between deep neck and trapezius muscle weakness and axial neck pain following cervical spine surgery [120,121]. Therefore, postoperative muscle strengthening is essential for improved outcomes [122,123]. Isometric exercises of the neck and trapezius muscles performed early after cervical spine surgery not only have muscle hypertrophy effects but also improve local blood circulation, with consequent favorable effects on muscle swelling and pain sensitization at the local surgical site [124]. The general neck and shoulder muscle strengths are shown. (Figure 3).

### 8.2. Hand Dexterity Movement Exercises

**Figure 4:**
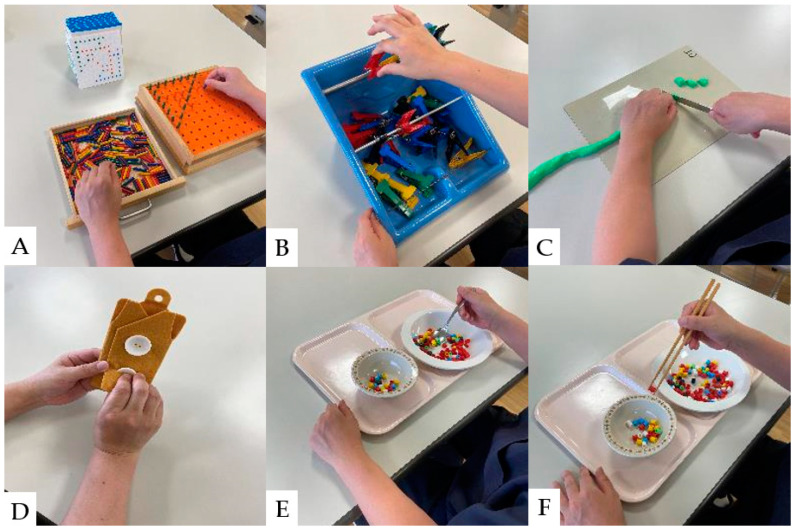
Hand dexterity movement exercises: (**A**): Pinching action with pegboard. (**B**): Pinch Power Strengthening Exercise. (**C**): Cutting exercise using a knife, (**D**): Buttoning practice. (**E**): Hand dexterity movement exercises using a spoon. (**F**): Hand dexterity movement exercises using chopsticks.

Hand sensory disturbances, hand dexterity disorders, and intrinsic hand muscle weakness are the initial clinical manifestations of CSM [116,117]. Hand dexterity disorders are closely associated with the severity of CSM [125]. A study of hand dexterity movement exercise in monkeys with artificially injured spinal cords revealed that hand dexterity improved when exercise was performed early in the injury [126]. In recent years, hand dexterity has improved with interventions combining general rehabilitation and electrical neuromodulation in patients with SCI [127,128]. To improve hand dexterity, tasks similar to activities of daily living should be adopted into the rehabilitation program. The results of conventional hand dexterity movement exercises are shown in Figure 4.

### 8.3. Neural Mobilizations (NM)

**Figure 5:**
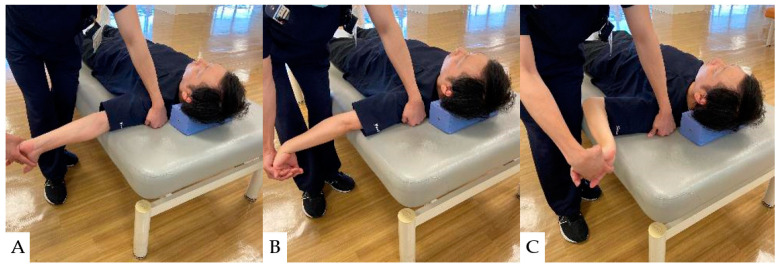
Neural Mobilization (NM): (**A**): Neural Mobilization of the median nerve area (C5~7), (**B**): Neural Mobilization of the radial nerve area (C6~8), (**C**): Neural Mobilization of the ulnar nerve area (C8~Th1).

Neural mobilizations (NMs) are interventions such as exercise and manual techniques intended to directly or indirectly affect neural tissue in conditions with signs of neural involvement or neural mechano-sensitivity [129,130]. NM effectively improves upper extremity pain and quality of life in patients with cervical radiculopathy [131,132]. Their mechanism of action is thought to involve affecting the axoplasmic flow movement of the nerve [133]. The nerve microcirculation may be affected by alterations in nervous system pressure and reductions in intraneural edema [134]. NM can also reduce the excitability of dorsal horn cells [135]; thus, it has been proven effective in enhancing upper extremity pain relief and quality of life in patients with cervical radiculopathy [131,132]. The specific NM techniques used for upper extremity pain in patients with cervical spine disease are illustrated in Figure 5.

### 8.4. Balance Ability Exercise

**Figure 6:**
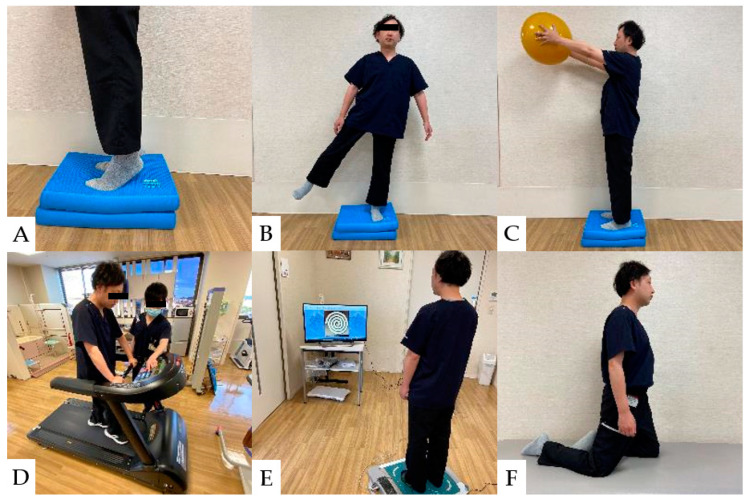
Balance ability exercise: (**A**): Stand on balance cushions and raise heels. (**B**): Stand with one foot on balance cushions and abduct the contralateral lower leg. (**C**): Stand on balance cushions and raise the ball. (**D**): Walk on a treadmill and react to changes in speed. (**E**): Center of gravity movement exercise using a TV game. (**F**): Knee walking on a platform. (Used for patients at high risk of falling).

The incidence of falls and fall-related fractures is more significant in CSM patients than in healthy adults and partially improves after spinal decompression surgery [136,137,138,139]. However, sensory deficits and impaired balance generally persist after cervical spine surgery [140]. In the balance assessment, the most difficult items for CSM patients were using the brief BESTest, hip/trunk lateral strength, standing on one leg, compensatory step correction, standing on foam, and eyes closed [91]. Rehabilitation for balance disorders in patients with CSM should improve postural control, lower limb strength, and lower limb muscle response to disturbances [141,142,143]. Balance ability exercise after CSM surgery is shown (Figure 6).

## 9. Virtual Reality Technology for the Rehabilitation

Virtual reality (VR) technology is increasingly being utilized for rehabilitation purposes, particularly in cases of cervical spinal cord disease [144]. Research using neuroimaging techniques has demonstrated that virtual reality can modify neural connections in various brain regions, including the primary sensory-motor cortex, supplementary motor area (SMA), cerebellum, precentral gyrus, and both ipsilateral and contralateral marginal gyri. This modification occurs through the integration of visual, auditory, and tactile feedback [145]. VR technology creates a lifelike environment that offers participants a secure and engaging platform for learning. The effectiveness of motor function restoration is directly related to the extent of neural network reorganization induced by the VR intervention. The recovery of neurological function in CSM patients has been shown to depend on both spinal cord compression and injury and reorganization or plasticity of brain function [146]. VR technology is being applied to facilitate rehabilitation through brain remodeling [147,148]. Upper extremity exercise with VR has yielded better results in SCI patients than conventional therapy [149], significantly improving muscle strength, gait, balance ability, and WISCI-II [150]. VR therapy stimulates patient attention and motivation, making the intervention more effective than traditional physical therapy [151]. However, the effect of rehabilitation via VR after CSM surgery is not yet apparent [144], and further research is needed to verify its effectiveness.

## 10. Rehabilitation for Postoperative Complications of Cervical Spine Surgery

### 10.1. Postoperative C5 Palsy

A frequent complication following cervical spine surgery is C5 palsy, which affects roughly 5.6% of patients [152]. This condition manifests as weakness in the deltoid and/or biceps muscles, either on one side or both [153]. Typically, C5 paralysis emerges within two weeks post-surgery and is temporary, usually resolving within six months [152,153]. However, the defect may sometimes persist, affecting 15% to 19% of patients [154]. Postoperative C5 palsy can lead to decreased patient satisfaction and reduced quality of life [155].

### 10.2. Physical Therapy after C5 Palsy

Regarding physical therapy, the first step is to evaluate the power of the deltoid and biceps muscles via the manual muscle test (MMT), (Table 5) [155]. The physiotherapy program is then tailored to the results of the MMT evaluation, with resistance exercise for MMT 4, active exercise for MMT 3, active assisted exercise for MMT 2, functional electrical stimulation for MMT 1, and electric muscle stimulation for MMT 0. Recently, robotic technology has been used to treat C5 palsy [156]. A study by Kubota et al. revealed that during physical therapy for C5 palsy via the hybrid assistive limb (HAL), patients with MMT grades 1 to 2 improved from grade 3 to 4, and the shoulder abduction angle improved from 36.4° to 140.7° [157]. While treating C5 palsy via robotic technology can reduce trick motion and promote normal muscle activity, further validation is needed [156].

### 10.3. Postoperative Dysphagia and Dyspnea

Postoperative dysphagia and dyspnea following cervical spine surgery are significant complications [158]. In posterior surgery, these complications are more common after occupational-cervical fixation (OCF) [159]. In severe cases, revision surgery is mandatory [160]. Prolonged intermediate dysphagia after cervical spine surgery can lead to psychological problems such as depression, anxiety, and other issues that decrease quality of life [161]. Therefore, early remediation is needed. Although the effectiveness of rehabilitation for postoperative dysphagia is not currently apparent, several commonly used approaches are described below.

### 10.4. Physiotherapy for Dysphagia after Anterior Fixation

**Figure 7:**
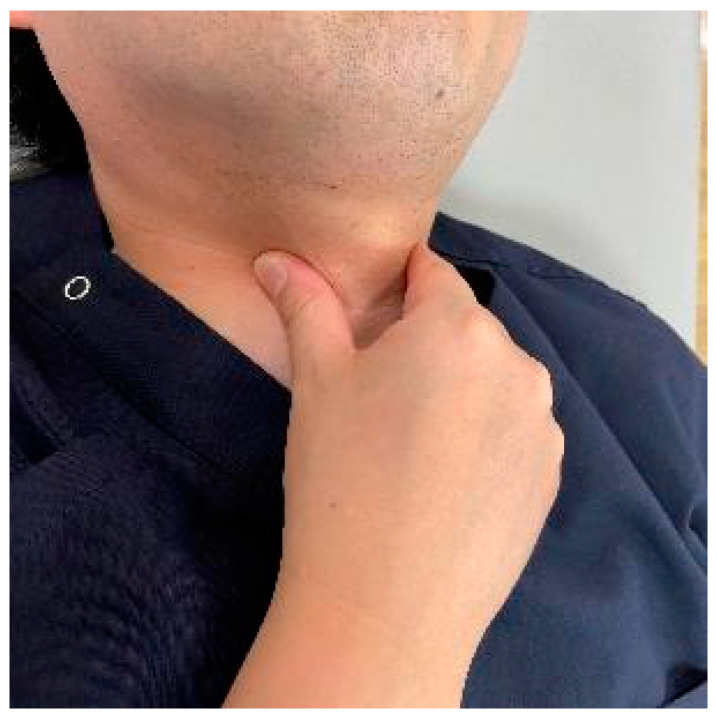
The manual tracheal retraction exercise (TRE).

**Figure 8:**
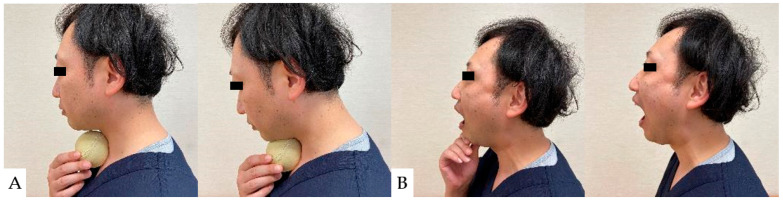
Suprahyoid muscle exercises: (**A**): Chin tack exercise, (**B**): Jaw opening exercise.

As a preoperative exercise, manual tracheal retraction exercise (TRE) is useful for preventing dysphagia (Figure 7). TRE has been shown to improve the flexibility of the tracheoesophageal sheath, reduce intraoperative retractor pressure, and reduce local and surrounding tissue damage [162]. It also reduced dysphagia more with multilevel anterior surgery [162]. For postoperative dysphagia, consideration should be given not to stress the cervical spine. Suprahyoid muscle exercises, including chin tuck and jaw opening exercises, are recommended (Figure 8) [163,164].

## 11. Physical Therapy for Cervical Cord Injury (CCI)

**Figure 9:**
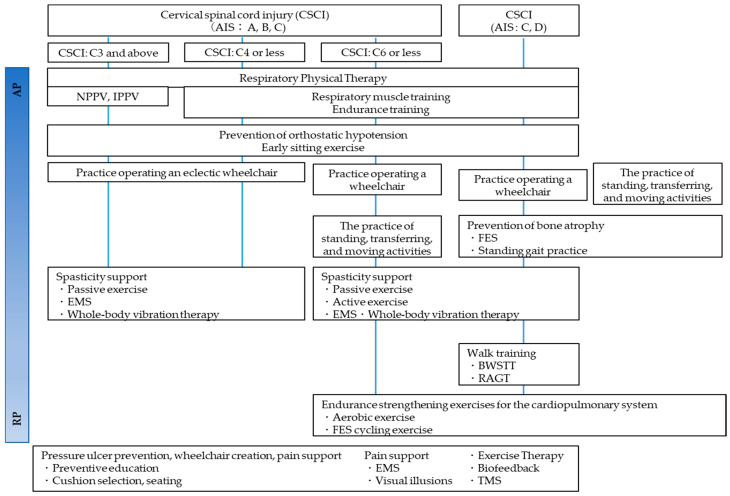
Physical therapy algorithm from Acute phase to Recovery phase [166].

**Figure 10:**
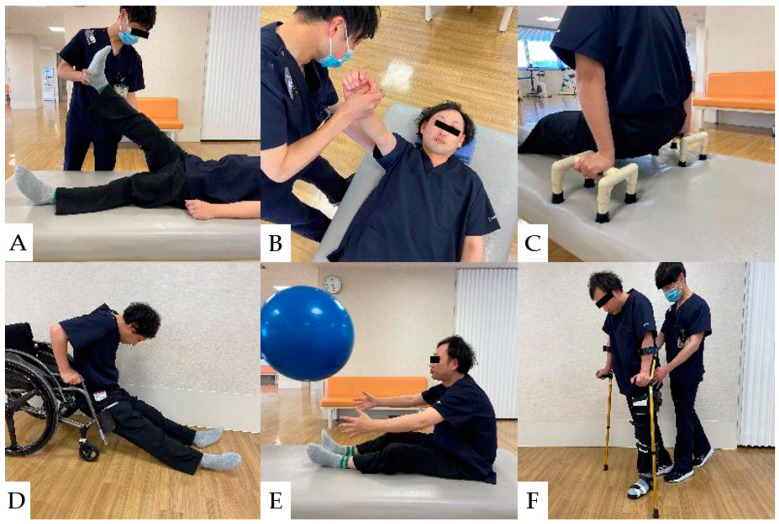
Physical Therapy for CCI Patients: (**A**): Stretching the hamstrings. (**B**): Strengthening exercises for the serratus anterior muscle. (**C**): Push-ups to Prevent Pressure Ulcers. (**D**): Floor-to-wheelchair transfers. (**E**): Strengthening of trunk muscles for stabilization of sitting position. (**F**): Gait practice with a cane and lower limb orthosis.

Approximately two months after injury is regarded as the acute phase of CCI. During this phase, the patient’s neurological status will be stabilized, and the goal of rehabilitation at such intervals is to prevent sedimentary pneumonia (respiratory complications), pressure ulcers, and orthostatic hypotension that may occur long-term [165]. After the acute phase, the most crucial key in the recovery phase is to set the rehabilitation goal for both complete and incomplete paraplegic patients. Several goals include free ambulation, gait with support, and wheelchair drive. For social mobility, the individual should be capable of traversing 50 m independently or with mobility devices [165]. Rehabilitation protocols and general physical therapy are shown from the acute postoperative phase of CCI to the recovery phase [166] (Figure 9 and Figure 10).

### 11.1. Acute Phase of CCI

#### 11.1.1. Pneumonia

Respiratory issues are a significant cause of illness and death in individuals with acute CCI [167]. Damage to the phrenic nerve can occur from injuries above the C4 level, necessitating ventilator support. Even injuries below C5 can significantly weaken respiratory muscles, including the intercostals and abdominal muscles, which may compromise breathing function [168]. Effective secretion management is crucial in caring for patients with CCI to avoid complications like mucous plugs, atelectasis, and pneumonia. Percussion, vibration, or assisted suctioning can facilitate secretion mobilization [169]. Individuals with CCI may experience improvements in respiratory function through respiratory muscle training [170].

#### 11.1.2. Pressure Sore

Patients with CCI are highly susceptible to pressure sores due to immobility, a lack of sensation, and other physiological changes, which can lead to skin breakdown and delay rehabilitation [171]. Those in lateral decubitus, sitting, and transferring to wheelchairs with full-assistance positions are at high risk of developing pressure sores [172,173]. The guidelines recommend turning or repositioning individuals with CCI every two hours during the acute rehabilitation phase and placing them on a pressure-reducing device [174].

#### 11.1.3. Low Blood Pressure

**Figure 11:**
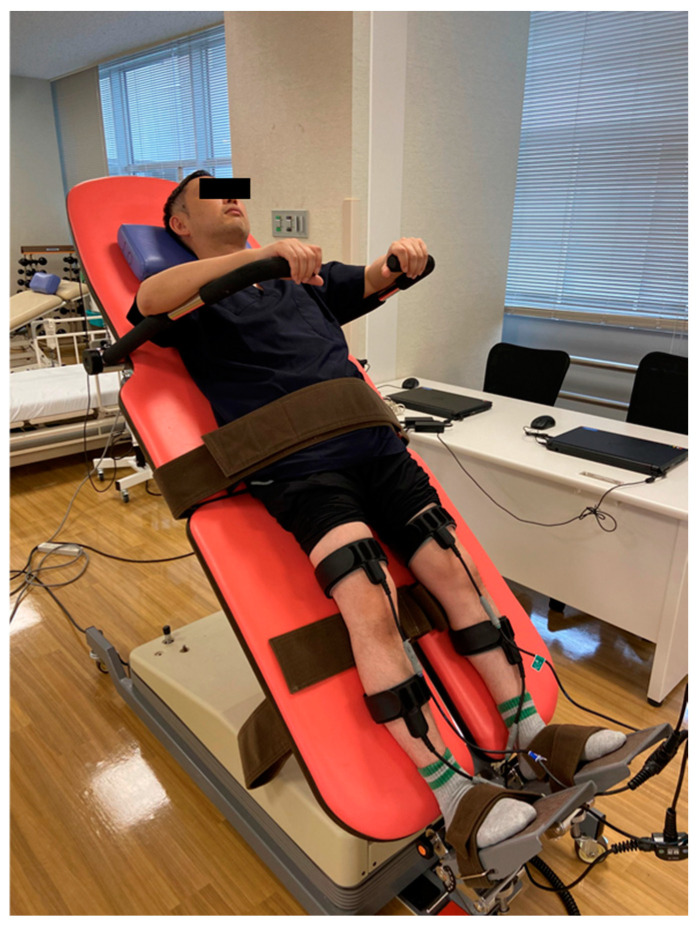
Treatment of OH using tilt table and EMS.

Patients with CCI often experience unstable blood pressure, which can result in frequent episodes of low blood pressure or orthostatic hypotension [175]. A tilt table can be helpful for patients with orthostatic hypotension, beginning from a 45-degree angle for 30 min per day, with the angle gradually increasing based on the patient’s complaints or condition [165]. During episodes of orthostatic hypotension, various non-pharmacological approaches can prove beneficial. These include applying compression and pressure to the abdomen, performing upper body exercises, utilizing functional electrical stimulation (FES) on the legs, and employing biofeedback techniques [176,177,178,179,180] (Figure 11).

### 11.2. Recovery Phase of CCI

#### 11.2.1. Goal of SCI Rehabilitation

The neurological level of injury (NLI) and paralysis severity are significant predictors of walking independence in individuals with CCI. An international standardized method developed by ASIA is widely employed for neurological assessment [181]. The ASIA Impairment Scale (AIS) is used to evaluate the severity of paralysis at 72 h or 1-month postinjury and can predict AIS at one-year postinjury [182]. In addition to motor function, pain perception and age at injury correlate with the ability to walk with the CCI [183,184]. Age at CCI injury and motor and tactile scores at the L3 and S1 levels within 15 days of injury foretell the acquisition of indoor walking one year after injury [185]. To predict ADLs that can be acquired by patients with CCIs, treatment programs should be designed based on appropriate prognostic predictions, and rehabilitation should be practiced as needed (Table 6).

#### 11.2.2. Sitting Training

Once the patient’s general condition is stabilized, sitting exercises are initiated. For CCI patients, stabilization of the sitting balance stabilizes the wheelchair sitting position and increases the standard of living [186]. Furthermore, improving the sitting posture of CCI patients is one of the primary objectives of rehabilitation because it enhances life satisfaction [187]. Rehabilitation to improve balance ability in CCI patients includes seated balance training [188], balance training on an unstable mat [189], and virtual reality exercise [190]. Seated balance exercises necessitate interventions focusing on sitting and postural control during daily upper extremity movements.

#### 11.2.3. Wheelchair Training

The goal of rehabilitation for CCI patients in the recovery phase focuses on acquiring mobility. Therefore, the initial stage of mobility acquisition in CCI rehabilitation involves learning wheelchair skills [191]. The wheelchair is the most effective device that not only enables mobility but also gains the freedom to participate in the community [192]. Upper extremity and trunk muscle strength are crucial for wheelchair propulsion and wheelchair-to-bed/floor transfer [193,194]. Wheelchair maneuver acquisition is related to life satisfaction [195]. Rehabilitation to gain wheelchair control includes a wheelchair skills training program [196], which improves sitting function and wheelchair control skills [197,198].

#### 11.2.4. Standing Training

CCI patients have worse standing balance and are at greater risk of falling [199,200]. CCI patients decrease their activity level due to fear of falling [201]. CCI patients’ standing balance control depends on visual input [199]. Therefore, balance exercises with visual feedback help CCI patients achieve stable standing. Balance exercises include visual feedback balance training (VFBT) to improve postural balance control in CCI patients [202]. Recently, balance exercises combining VFBT and FES have been effective for standing balance in patients with CCI [203].

#### 11.2.5. Gait Training

**Figure 12:**
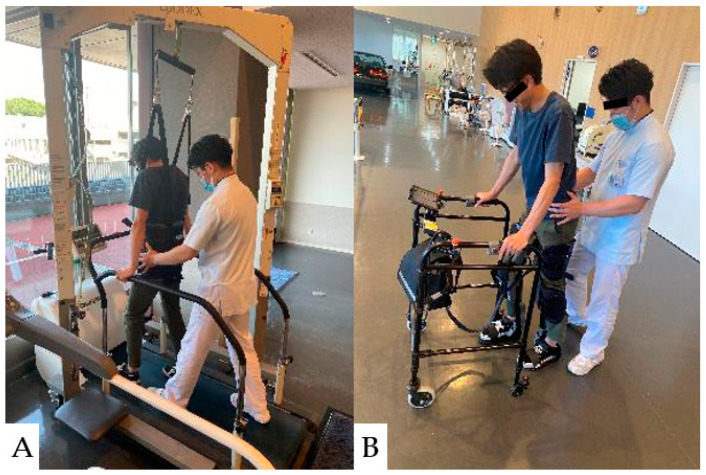
Gait practice for SCI patients using body weight-bearing treadmill training (BWSTT) and robot-assisted gait training (RAGT). (**A**): Body weight-supported treadmill training, (**B**): Body weight-supported training with a walker.

Body weight-supported treadmill training (BWSTT) and robot-assisted gait training (RAGT) are used for gait practice in patients with CCI (Figure 12). In the BWSTT, gait training is initiated early and uses a symmetrical gait pattern of the lower extremities to improve step and balance [204]. However, reproducing a normal gait pattern with manual BWSTT requires the assistance of multiple therapists. Therapist-assisted gait practice is physically demanding and exhausting; therefore, the RAGT was introduced [205].

RAGT has advantages such as increasing the intensity and total duration of training while maintaining the gait pattern. Compared with the BWSTT, the RAGT allows patients with severe CCIs to begin gait training earlier, reduces physical therapist effort, and increases gait duration and intensity [206]. The reported effects of BWSTT and RAGT on patients with CCI are shown in Table 7.

## 12. Postoperative Rehabilitation of Cervical Tumors

In recent years, advancements have been made in research on rehabilitating cancer patients undergoing treatment [213]. The emphasis is placed on ADLs and quality of life in the remainder of a cancer patient’s life to ensure long-term survival [214]. CCT causes pain and paralysis [215] due to spinal cord compression, which can also alter the structure of the spine, resulting in spinal instability. The treatment of CCT depends on the spine’s stability, neurological status, and the presence or absence of pain. Different treatment options, including surgical and non-surgical, can be combined with rehabilitation to relieve symptoms, improve quality of life, and increase functional independence in patients with malignant spinal cord compression [216].

The Tokuhashi score has been utilized to determine treatment strategies for patients with CCT [217], and it is useful as a scoring system for predicting life expectancy in patients with CCI [218] (Table 8). The goals of rehabilitation for patients with CCT are similar to those for patients with traumatic SCI: providing patient and caregiver education, improving mobility and safety, maximizing independence, and facilitating safe discharge to the community [219]. In addition, based on the severity of paralysis and the CCI [220], improvements in ambulation are associated with improved life expectancy. During inpatient rehabilitation, 65% of CCT patients are discharged home, significantly improving FIM during hospital discharge [221]. Gait practice for CCT patients with RAGT improves gait ataxia [222], and rehabilitation results in less pain and depression [223].

Patients with CCI may experience further complications due to primary cancer or metastatic disease [224,225]. Patients with primary tumors who undergo an inpatient rehabilitation program have a median survival of 9.5 months, a 47.4% 1-year survival rate, and a 10.5% 5-year survival rate. In contrast, patients with metastatic disease have a median survival of 2.8 months, a 21.4% 1-year survival rate, and a 3.6% 5-year survival rate [225]. Survival rates vary depending on tumor pathology, with lung metastases having a 16% survival rate at 24 months and breasts having a 44% survival rate at 24 months [226].

Rehabilitation for patients with CCT should be extensive. The severity of NLI and CCI, life expectancy, and symptoms of the primary tumor should be considered to determine post-discharge goals. The following is a list of factors that may be important in rehabilitating CCT patients (Table 9).

## 13. Limitation

The limitation of this study is that it is a narrative review, which may introduce subjective bias in our perspectives and interpretations. It is also not a systematic review and may lack comprehensiveness and reproducibility. The number of databases used was limited, and the choice of keywords was only seven. We did not include reports with low-impact factors in this study, and we could not evaluate the quality of the included studies.

## 14. Conclusions

Rehabilitation of the cervical spine after surgery is essential for improving physical function and the ability to perform daily activities and enhance overall quality of life. The multifaceted rehabilitation process aims to restore mobility, improve functionality, and boost life quality. To evaluate and assess lumbar spine conditions, practitioners employ various methods, including physical therapy, cognitive–behavioral therapy, and activities of daily living, utilizing patient-reported outcomes and physical performance assessments. While current rehabilitative approaches heavily focus on strengthening muscles, the significance of spinal balance is frequently neglected. Therefore, giving equal attention to muscle strengthening and enhancing spinal balance following cervical spine surgical procedures is crucial.

## Figures and Tables

**Table 1:** History of Rehabilitation.

Year	Event
1914–1924	Blossoming of rehabilitation (mainly for occupational rehabilitation because of World War I) [16]
1935–1955	Spinal cord injury rehabilitation developed [17]
1944	The word “Rehabilitation” was defined by the British Council for Rehabilitation [18]
1968	Rehabilitation for ADL improvement by the World Health Organization (WHO) [19]
1977	Rehabilitation framework as a biopsychosocial model of illness [20]
1980s	Rehabilitation for QOL improvement, especially for elderly patients [21]
2016	Ontario protocol for traffic injury management [22]
2018	Combined treatments of physical and psychological treatments [23] New concept of psychological factors [24]

**Table 2:** Examples of postoperatively cervical spine rehabilitation.

Intervention	Definition	Example
Patient education and self-management [28,29]	Educate patients about their neck pain and how to reduce pain and suffering.Reduce mortality and morbidity after CCI and improve quality of life.	➢ How to deal with pain ➢ The importance of physical activity in pain reduction ➢ Mitigate pain flare-ups ➢ Step-by-step rehabilitation methods for return to routine work ➢ Prevention of complications (bedsores, urinary tract infections, etc.)
Early Exercise [30,31]	Preventing axial pain by strengthening cervical muscles and ROM exercises early after surgery. Strengthen respiratory muscles and prevent pneumonia after CCI.	➢ Stretching ➢ Muscle strengthening ➢ Endurance exercises ➢ Range of motion exercise
Manual therapies [32,33,34]	Manual therapies can relieve neck pain and radicular pain in the upper limbs.Massage Therapy Relieve stiffness and numbness after CCI.	➢ Myofascial release ➢ Neural mobilization ➢ Massage ➢ Traction
Electrical stimulation therapy [35,36]	Electrical stimulation therapy can improve pain, muscle activation, and coordination.	➢ Transcutaneous electrical nerve stimulation ➢ Functional electrical stimulation therapy
Body weight-supported gait training [37]	Body weight-supported gait training improves neural plasticity—the tendency of synapses and neural circuits to change in response to activity—by providing intensive locomotor gait training.	➢ Body weight-supported overground training ➢ Body weight-supported treadmill training ➢ Robot-assisted gait training

**Table 3:** The reported MCID for cervical spine surgery.

Study	PRO	Recommended MCID	Procedure	Diagnosis
Badhiwala [43]	PCS-36	4	Cervical decompression	Cervical myelopathy
	MCS-36	4		
Kato [44]	JOACMEQ Cervical spine function	2.5	Laminoplasty	Cervical myelopathy
	JOACMEQ Upper extremity function	13		
	JOACMEQ Lower extremity function	9.35		
	JOACMEQ Bladder function	7.7		
	JOACMEQ QOL	9.5		
Oshima [45]	COMI sum score	2.1	Not specified	Cervical degenerative disease
Carreon [46]	NDI	7.5	Cervical fusion	Cervical degenerative disease
	NRS neck	2.5		
	NRS arm	2.5		
Javeed [47]	DASH	−8		
Okano [48]	SWAL-QOL	−8	Anterior cervical discectomy and fusion	Cervical degenerative disease

PCS; physical component score, MCS; mental component score, JOACMEQ; Japanese Orthopedic Association Cervical Myelopathy questionnaire, COMI; core outcome measure index, NDI; Neck Disability Index, NRS; numerical rating score, DASH; Disabilities of Arm Shoulder and Hand, SWALL-QOL; Swallowing-related Quality of Life.

**Table 5:** Physiotherapy according to muscle strength in postoperative C5 palsy.

MMT Grade	Physiotherapy
0	Range of motion exercises Electric Muscle Stimulation, Robot
1	Range of motion exercises, Functional Electrical Stimulation
2	Range of motion exercises, Active assisted exercise
3	Active exercise
4	Resistance exercise

**Table 6:** The goal of CCI rehabilitation.

Residual Height	Main Muscle	Motor Function	Activity of Daily Living	Self-Help Devices and Orthotics
C2–C3	Sternocleidomastoid muscle	Head forward bending and rotation	Total support	Ventilator Electric wheelchair
C4	Transverse diaphragm Trapezius muscle	Head and neck movement Scapular elevation	Total support	Environment controller Lifter, mouse stick
C5	Deltoid muscle Biceps brachii muscle	Shoulder joint exercises Flexion–extension and rotation of the elbow joint	BFO and eating movements with orthotics and self-help devices	Wheelchair on flat ground Electric typewriter
C6	Pectoralis major muscle Extensor carpi radialis muscle	Shoulder joint adduction Extension of the wrist joint	Transferring (back and forth) possible, wheelchair-driven, turning over in bed, changing jackets	Tenodesis-synth print
C7	Triceps brachii muscle Flexor carpi radialis muscle	Elbow joint extension Palmar flexion of the wrist joint	Independent movement on the floor and transferring Independence in dressing Able to ride a bicycle	
C8–T1	Intrinsic muscles of the hand	Finger flexion	Independent of ADL in the wheelchair	

**Table 7:** Effect of BWSTT and RAGT on Patients With CCI.

Author	Intervention	Result
Walia [207]	BWSTT	Improve Standing Balance
Alajam [208]	BWSTT	Improve cardiovascular and pulmonary health
Alexeeva [209]	BWSTT	Improvement in maximal walking speed, muscle strength, and psychological well-being
Alashram [210]	RAGT	Improve gait speed, walking distance, strength, range of motion, and mobility
Gil-Agudo [211]	RAGT	Improved their walking independence as measured by the WISCI-II
Fang [212]	RAGT	Improve spasticity and walking ability

BWSTT: body weight-supported treadmill training, RAGT: robot-assisted gait training.

**Table 8:** Tokuhashi score [217].

Prognosis Parameter	Score
Patient condition	
Poor (performance status: 10–40%)	0
Moderate (performance status: 50–70%)	1
Good (performance status: 80–100%)	2
No. of bone metastases outside spine	
Poor (performance status: 10–40%	0
Moderate (performance status: 50–70%)	1
Good (performance status: 80–100%)	2
No. of bone metastases outside spine	
>2	0
1–2	1
0	2
Metastasis to major organs	
Nonremovable	0
Removable	1
None	2
Primary site	
Lung; osteosarcoma; stomach; bladder; esophagus; pancreas	0
Liver; gallbladder; unidentified	1
Other	2
Kidney; uterus	3
Rectum	4
Thyroid; breast; prostate; carcinoid tumor	5
Palsy	
Complete (Frankel A; B)	0
Incomplete (Frankel C; D)	1
None (Frankel E)	2

**Table 9:** Rehabilitation key after CCT surgery.

Intervention	Definition
Neurological Evaluation of Spinal Cord Injury	Assess neurological status and severity of spinal cord injury. As the severity of spinal cord injury increases, changes in improvement in neurologic function are scant.
Life expectancy	The prognosis varies greatly depending on the type of tumor, and evaluation is necessary to construct rehabilitation goals.
Symptoms of Primary Tumors	In the case of metastatic tumors, rehabilitation should be performed, considering the symptoms of the primary tumor.
Support for pain caused by osteolysis	Secondary health conditions can affect a patient’s ability to participate in rehabilitation and must be optimally managed. First, a comprehensive assessment and management of pain must be performed.
Social support at hospital discharge	The patient’s social support significantly impacts his or her plans for home after discharge and should be discussed from the beginning of rehabilitation.
